# Choice Bracketing revisited: Replication and extensions Registered Report of seven experiments reviewed in Read *et al*. (1999)

**DOI:** 10.1098/rsos.240687

**Published:** 2025-04-02

**Authors:** Chun Lam Wong, Gilad Feldman

**Affiliations:** ^1^ Department of Psychology, University of Hong Kong, Pok Fu Lam, Hong Kong

**Keywords:** choice bracketing, judgement and decision-making, registered report, replication, decision framing, joint versus separate evaluation

## Abstract

Choice partitioning refers to the phenomenon when the same choice set yields different decision-making behaviour when they are grouped into sets (broadly bracketed) or evaluated separately (narrowly bracketed). In a Registered Report experiment with a US sample recruited online through Prolific (*N* = 896), we conducted a replication of seven studies reviewed in Read *et al*. (Read *et al*. 1999 *J. Risk Uncertain*. **19**, 99 (doi:10.1023/A:1007879411489)). We concluded a mostly successful replication: out of the seven studies, we found support for six (Studies 1, 3, 4 and 6: Cramer’s *V* > 0.31; Studies 2 and 7: Cohen’s *d* > 0.29) and no empirical support for one (Study 5: Cramer’s *V* = 0.02). Extending the replication, we added new conditions in Studies 6 and 7, further expanding the manipulation’s scope range, yet failed to find any impact. In our replication, we came across many challenges, both conceptual and empirical, and we therefore call bracketing scholars to better define bracketing in relation to other phenomena in decision-making (joint versus separate mode, framing effects, mental accounting, etc.), with falsifiable hypotheses, examining overlap with other constructs, and clearer mapping between theory and empirics. Materials, data and code are available on: https://osf.io/vdqek/.

## 1. Background

Read *et al*. [1] seminal review summarized empirical studies on the ‘choice bracketing’ phenomenon. When choices are bundled together (broad bracketing), people have a stronger tendency to consider the consequences of all the options in the choice set compared with when these choices are presented as separate choices (narrow bracketing). One example used in their review paper was that smokers who smoke one cigarette every day (narrow bracketing) may not seem like a lot and thought to only have small negative health effects compared with the satisfaction obtained from smoking the cigarette. Yet, when the same decision is framed as having 365 cigarettes per year (broad bracketing), the aggregated health consequences from a year of smoking seem far more consequential than the momentary satisfaction from smoking, possibly shifting the smoker’s preferences away from having that one cigarette.

We conducted a close replication and extension Registered Report of the empirical demonstrations reviewed in Read *et al*. [1] with the following goals. Our first goal was to conduct an independent close replication of classic choice bracketing, outcome editing and choice partitioning experiments. Our second goal was to extend the target article’s design to examine if further broadening the bracket of a choice set enhances the effect of broad bracketing.

We begin by introducing the literature on choice bracketing and the chosen review article—Read *et al*. [1]. We then discuss our motivations for the current replication, outline our chosen studies for replication from the target article, the experimental designs, and our adaptations and extensions.

## 2. Choice Bracketing: Read *et al*. [1]

A choice can be thought of as a singular decision or can be seen as part of many similar repeating choices in a much larger choice set, within a combined choice bundle. A decision regarding a single choice considered alone may differ from a decision regarding the same choice when it is considered as part of a much broader scope and bundled choice set. For example, the decision to have a doughnut after work as ‘a little treat’ may seem enticing and reasonable as the expected pleasure may seem to outweigh the localized health consequences. Yet, if one considers all 260 single-doughnut eating choices one faces in a year together, it allows the decision-maker to consider the broader implications for one’s own health and shift the perceived trade-off between treat and health, and therefore the decision as to whether to have the doughnut or not.

Read *et al*. [1] reviewed studies demonstrating ‘choice bracketing’ effects—that when the choice sets are bundled (bracketed) to induce consideration of a larger set of hedonic consequences of a decision, it tends to induce more ‘optimal’ decision-making behaviour, in that it allows the person to consider broader implications and therefore align the singular choice better with own long-term goals and values. Inversely, consideration of a smaller set of consequences limits the ability to fully consider the utility of a singular decision.

In this literature, framing a choice individually or as a bundle is referred to as ‘choice bracketing’. ‘Narrow bracketing’ refers to framing options as separate decisions to focus the consideration on the local consequences of a behaviour (i.e. eating a doughnut after work today), and ‘broad bracketing’ refers to framing decisions as part of a larger choice set to encourage consideration of broader consequences of a behaviour (i.e. eating 260 doughnuts for a year) [1].

The tendency to bracket narrowly and perceive choice sets as separate individual decisions might be explained by cognitive load, given that broad bracketing is more cognitively taxing than narrow bracketing. When considering a larger choice set, it is cognitively demanding to simulate and compute the estimated results of all choice combinations as each additional decision is added to the bundle. For example, Brown *et al*. [2] investigated the reluctance of retired individuals to trade annuities and showed that one’s cognitive ability is negatively associated with their tendency to engage in broad bracketing (refusing to sell their annuities on the onset of their retirement by considering the complex cost–benefit analysis of their decision), showing the impact of the cognitively loading nature on inhibiting broad bracketing decision-making.

Mental accounting heuristics that guide us to fragment and categorize objects, behaviours and events might also be a source of narrow bracketing [3,4]. For example, a person may categorize eating a handful of nuts at 15.00 as ‘snacking’, an indulgent behaviour, and might therefore decide against it, yet perceive adding a handful of nuts to a Caesar salad at noon as simply part of ‘eating lunch’, a normal and accepted behaviour. These two behaviours are the same, aside from their context and the time frame of when they were considered. A problem is often perceived and processed as it is presented: if the choice is presented alone, then it is more likely to be evaluated as a separate choice, yet if the choice is presented as part of a bundle, then it is more likely to be evaluated as part of the greater bundle. Moreover, choice is affected by which bundle, or mental account, the choice is presented as part of.

## 3. Choice of article for replication: Read *et al*. [1]

We embarked on a replication and extension Registered Report of Read *et al*. [1]. We aimed to revisit the phenomenon to examine the reproducibility and replicability of the findings with an independent pre-registered close and well-powered replication and extension. This follows the recent growing recognition of the importance of reproducibility and replicability in psychological science (e.g. [5,6]).

We chose Read *et al*. [1] based on several factors: the potential for improvements in methodology and/or theory, its academic and practical impact, mixed findings in the literature and the absence of direct replications.

First, we consider it a promising direction to conduct a comprehensive empirical test with a replication of many similar studies that were reviewed in a single review article. We previously conducted systematic replications of seminal review papers (Au & Feldman [7]; Li & Feldman [8]; Hong & Feldman [9]) and of papers reviewing a large number of empirical studies on a single phenomenon [10]. We see much value in those, especially as for the target phenomenon they allow for: (i) a comprehensive empirical test of many different methods, and (ii) the examination of in-person consistency in responding to many decision situations.

Second, the simple and straightforward designs of the choice bracketing empirical studies reviewed in this paper allowed us to easily add extensions and tests that help gain additional insights regarding the nature of bracketing effects. For example, Read *et al*. [1] mostly reviewed bracketing contrasting two bracketing options, and a simple extension is to add a control condition or further broaden or narrow one of these options.

Third, as we revisited and analysed the studies in the review article, we realized that some studies were in need of revisiting. Some descriptions were incomplete, and some reporting of the analyses in some of the studies was unclear, such as only reporting the descriptive data without testing. Reconstructing the materials, rethinking the design and statistical analyses, and revisiting the experiments with fuller reporting may help future studies in this domain.

Fourth, the article has had an impact on scholarly research in the areas of social psychology, judgement and decision-making, and behavioural economics. At the time of writing (October 2024), there were 1166 Google Scholar citations of the article and many important associated and follow-up theoretical and empirical articles, such as Thaler’s [11] work on the components and formation of mental accounting, and Barberis and Huang’s [12] study on the importance of choice bracketing in stock market and gamble participation.

Finally, to our knowledge, there are currently no published comprehensive direct replications covering all the reviewed paradigms and empirical studies in Read *et al*. [1]. One direct pre-registered replication of one of the reviewed paradigms in the target article is by Vonasch *et al*. [13] showing a mostly successful replication of Hsee [14], comparing joint versus separate evaluations in a similar way to the reviewed Hsee [15]. They found similar findings, though with added extensions suggesting potential for more nuanced findings. Several comprehensive empirical studies reran some versions of some of the described empirical studies in Read *et al*. [1], generally concluding support for instances of bracketing in their contexts, and identifying mechanisms and moderators (Moher & Koehler [16]; Webb & Shu [4]; Rabin & Weizsäcker [17]).

There are also successful conceptual replications of some of the empirical studies. Chang *et al*. [18] examined joint versus separate evaluation on the salary of candidates and Jolles *et al*. [19] examined the impact of joint versus separate evaluation on age and gender diversity. Both were successful replications showing the impact of bracketing on decision-making in companies’ hiring decisions. Felso and Soetevent (2014) [20] showed the impact of bracketing on gift-card spending behaviour, finding that broad bracketing conditions encourage more flexible spending behaviour. Hadar *et al*. [21] also provided an example for the impact of bracketing on utility maximization in experience-based choices.

Examining the determinants of choice bracketing, Brown and colleagues [2] investigated how increasing the complexity of annuity choices can encourage narrow bracketing when valuing annuities.

The follow-up conceptual replications and novel studies on bracketing effects suggest support for the phenomenon, with some moderators, and yet there is a lack of pre-registered (/Registered Report) well-powered studies that would directly and closely replicate the core seminal classic experiments that were reviewed by Read *et al*. [1].

## 4. Read *et al*. [1]: Design, hypotheses and findings

Out of the 18 studies that Read *et al*. [1] reviewed in the article, we focused the replication on seven, with the remaining 11 studies deemed not feasible (e.g. field studies) or substantially overlapping with the chosen studies. For easier reference, we renamed those into Studies 1 to 7, and summarized the selected studies’ hypotheses in table 1. The complete list of the studies reviewed in Read *et al*. [1] are listed in table 2, indicating our reasons for exclusions from the studies not chosen for replication.

**Table 1.** Summary of hypotheses of the reviewed empirical studies in Read *et al*. [1] and our extensions.

Study	Section in target	Label in target	Scenario	Hypothesis
**Replication**				
1	2.2	Choice bracketing	Choices between risk seeking and risk-averse options.	People tend to choose higher value choices in integrated one-choice presentation mode compared with two-choice presentation mode.
	2.2	Outcome Eediting		People tend to choose higher value choices in segregated one-choice presentation mode compared with two-choice presentation mode.
2	2.3	Joint versus separate evaluation of alternatives	Salary evaluation	In comparing candidates on experience and grades, joint evaluation mode shifts evaluations towards valuing experience over grades, compared with separate evaluation mode.
3	3.1	Scheduling future experiences	Scheduling two activity categories (gardening and reading) with one pleasant activity and one unpleasant activity in each, over a course of 2 weeks.	When scheduling one pleasant activity and one unpleasant activity from two activity categories (gardening and reading), those scheduling in joint evaluation mode (all options presented together) tend to diversify the pleasant and unpleasant experiences, compared with separate evaluation mode (choosing separately for each activity category).
4	3.2	Adding-up effect	Taking a bet of small vs. large value	In a choice between an uncertain bet and a certain option, of similar value, the larger the sums the more likely people are to choose the certain option.
5	3.4	Tradeoffs across choices	Making separate bets versus joint repeated bets	Joint presentation of repeating bets decreases the likelihood to accept the first bet compared with separated presentation of repeating bets.
6	4	Determinants of bracketing - cognitive inertia	Deciding on whether to purchase a desired concert ticket given a mental allocation of a budget for a predefined time frame.	The likelihood of purchasing a desired concert ticket is lower if one perceives purchasing the ticket as exceeding a mental allocation of a budget for a predefined time frame.
7	4	Pre-existing heuristics	Ordering free pudding dessert for 7 days event	The number of free desserts ordered for a 7 days event is lower when ordering the free desserts is done in joint mode for all 7days together in one page, than in joint mode for weekdays together and then weekend together, and both are lower compared with a separate display of ordering for each day separately on a different page.
**Extensions**				
Study 6	N/A	Broadening the bracketing frame	Decide to go to a concert or not (fiscal period lengthened to yearly)	Broader bracketing encourages higher-value choices.
Study 7	N/A	Narrowing the bracketing frame	Dessert-eating planning (week presentation narrowed to daily decisions)	Narrower bracketing discourages higher-value choices.

**Table 2.** Read *et al*. [1]: studies for inclusion and reasons for exclusion.

experiment (section (sub-section) author (year))	replication decision	reason for exclusion
2 (2.1) Tversky & Kahneman [22]	Replicated: Study 1	
2 (2.2) Read *et al*. [1]	Replicated: Study 1	
2 (2.3) Hsee [15]	Replicated: Study 2	
3 (3.1—Diversity) Simonson [23]	Not replicated	Field experiment
3 (3.1—Diversity) Simonson and Winer [24]	Not replicated	Field experiment
3 (3.1—Diversity) Read and Loewenstein [25]	Not replicated	Field experiment
3 (3.1—Scheduling future experiences…) Loewenstein and Prelec [26]	Not replicated	Overlaps with Study 4 (3 (3.1—Scheduling future experiences…)) in Read *et al*. [1], which provided more information regarding the experimental design.
3 (3.1—Scheduling future experiences…) Read *et al*. [1]	Replicated: Study 3	
3 (3.1—Risk aggregation) Thaler [11]	Not replicated	Not an experiment. Anecdotal descriptive evidence based on discussions with executives and CEOs, a sample we have no access to.
3 (3.1—Risk aggregation) Benartzi and Thaler [27]	Not replicated	Field experiment, focused on myopic loss aversion, a separate though related phenomenon.
3 (3.2) Read *et al*. [1]	Replicated: Study 4	
3 (3.3—Habit formation) Kudadjie-Gyamfi and Rachlin [28]	Not replicated	Reaction time experiment, not possible with the other studies and using our surveying platform (Qualtrics).
*3 (3.4—Trade-offs between labour and leisure)* Camerer *et al. [**29**]*	Not replicated	Field experiment
3 (3.4—Trade-offs across purchase categories) O’Curry [30] *&* Heath & Soll [31]	Not replicated	Overlaps with Study 5 (3[3.4—Trade-offs across purchase categories] Read *et al*. [1]), which provided more information regarding the experimental design.
3 (3.4—Trade-offs across purchase categories) Read *et al*. [1]	Replicated: Study 5	
4 (Cognitive inertia) Redelmeier & Tversky [32]	Not replicated	Overlaps with Study 6 (4(Cognitive inertia) Read *et al*. [1]), which provided more information regarding the experimental design.
4 (Cognitive inertia) Read *et al*. [1]	Replicated: Study 6	
4 (Pre-existing heuristics) Read *et al*. [1]	Replicated: Study 7	

### 4.1. Study 1 (Choice Bracketing and outcome editing)

Study 1 included two sections (2.1 and 2.2), both offering a manipulation of a classic vignette by Tversky & Kahneman [22]. We combined the original and the two new versions from the two sections into a singular unified experimental design contrasting the three versions against each other. We summarized the study design in table 3.

**Table 3.** Study 1 (choice bracketing and outcome editing): options and study design.

combos	choice	label	meaning
Single choices	A	Choice I: Gain-Certain	100% gain of $240
	B	Choice I: Gain-Risky	25% chance to gain $1000 75% chance to gain nothing
	C	Choice II: Loss-Certain	100% loss of $750
	D	Choice II: Loss-Risky	75% chance to lose $1000 25% chance to lose nothing
A+D	AD-I	Integrated display	25% chance to gain $240 75% chance to lose $760
	AD-S	Segregated display	100% gain $240 and 75% chance to lose $1000 25% chance to lose nothing
B+C	BC-I	Integrated display	25% chance to gain $250 75% chance to lose $750
	BC-S	Segregated display	100% lose $750 and 25% chance to gain $1000 75% chance to lose nothing
Study design (between-subject):			
Single-choice segregated outcomes (1a)	Single-choice integrated outcomes (1b)	Two-choice display	
Choose either A 100% gain of $240; combined with a 75% chance to lose $1000 and 25% chance to lose nothing or a 100% loss of $750; combined with a 75% chance to gain nothing and 25% chance to gain $1000	Choose either 25% chance to gain $240; 75% chance to lose $760 or 25% chance to gain $250; 75% chance to lose $750	Two sets (random order): Set X: Choose either 100% gain of $240 or 25% chance to gain $1000; 75% chance to gain nothing Set Y: Choose either 100% loss of $750 or 75% chance to lose $1000; 25% chance to lose nothing	
Dependent variable: Participants choosing AD (or A+D) versus BC (or B+C)			
‘Imagine that you face the following pair of concurrent decisions. First, examine both decisions, then indicate the option you prefer’			

Sources in target article: Sections 2.1 Choice Bracketing, p.174 and 2.2 Outcome Editing, p.175.

The experiment builds on the classic two-choice loss–gain framing effect paradigm by Tversky & Kahneman [22] with two decisions between two options (A or B; C or D). The options offer numerical bets with a certain possibility to gain or to lose money. The first step is a decision between these two choices: (A) 100% gain of $240, or (B) 25% chance to gain $1000 and 75% chance to gain nothing. The second step is a decision between: (C) 100% loss of $750, or (D) 75% chance to lose $1000 and 25% chance to lose nothing.

In the first section (which they termed ‘choice bracketing’), they added a condition in which the two-step decisions were combined into a single-step. A was combined with D resulting in: AD—‘a 100% gain of $240; combined with a 75% chance to lose $1000 and 25% chance to lose nothing’. B was combined with C resulting in: BC—‘a 100% loss of $750; combined with a 75% chance to gain nothing and 25% chance to gain $1000’.

In the second section (which they termed ‘outcome editing’), they added a condition in which the two-step decisions were again combined into a single-step, yet this time instead of the ‘segregated’ form in the first second, they used ‘integrated’ form integrating the information from all probabilities into unified information about the probabilities of loss and gain. Meaning, that A and D were integrated into ‘25% chance to gain $240; 75% chance to lose $760’ and B and C were integrated into ‘25% chance to gain $250; 75% chance to lose $750’.

Their findings across both sections were that people tend to choose the higher utility choices in a single choice, both segregated and integrated, compared with the original two-choice decision by Tversky & Kahneman [22]. To allow for the comparison of the two-choice with the single choice, we calculated the net value of the combined choices in the two-choice, and compared that with the net-value of the single choice.

### 4.2. Study 2 (Joint versus separate evaluation of alternatives; Hsee [15])

In Study 2, participants evaluated two candidates J and S and indicated their suggested annual salary offer for that candidate. In the joint evaluation condition, both candidates’ salaries were evaluated together, whereas in the individual evaluation conditions, the participants only evaluated one of the two candidates. We summarized the study design in table 4.

**Table 4.** Study 2 (joint versus separate evaluation of alternatives [15]): options and study design.

Condition	Description	
More experience, lower GPA	Candidate J: 70 similar coding projects; 3.0/5 GPA	
Less experience, higher GPA	Candidate S: 10 similar coding projects; 4.9/5 GPA	
Separate evaluation: candidate J only	Separate evaluation: candidate S only	Joint evaluation: both candidates J and S
More experience, lower GPA 70 similar coding projects; 3.0/5 GPA	Less experience, higher GPA 10 similar coding projects; 4.9/5 GPA	Candidates presented side by side, and rated together. 70 similar coding projects; 3.0/5 GPA 10 similar coding projects; 4.9/5 GPA
Dependent variable: offered salary for the candidate		
‘Assume that you are the owner of a consulting firm. You are looking for a computer programmer who could write in a special computer language: KY language. The two candidates, who are both new graduates, differ on two attributes: experience with the KY language and undergraduate GPA (on a 5-point scale).’		
Separate evaluation condition question set: ‘How much will you pay for the salary of candidate S/J?’ joint evaluation condition question set: ‘How much will you pay for the salary of each candidate? You can choose different salaries.’		
[Candidate(s) then presented according to the assigned condition]		
Scale: 50,000 or less; 60,000; 70,000; 80,000; 90,000; 100,000; 110,000; 120,000; 130,000; 140,000;150,000 or more		

Note. Salaries were adjusted to match the inflation since approximately 1999. Source: CPI inflation calculator

### 4.3. Study 3 (Scheduling future experiences)

In Study 3, participants are tasked to schedule two pairs of two activities over two weekends, and in each pair there was one alleged ‘desirable’ experience (e.g. planting flower bulbs), and one alleged ‘undesirable experience’ (e.g. weeding). In the joint evaluation condition, all four choices were presented in one set, whereas in the separate evaluation condition, the choices are presented one pair at a time. We summarized the study design in table 5.

**Table 5.** Study 3 (scheduling future experiences): options and study design.

Variables	Expression
A (gardening—unpleasant)	Do weeding for 2 h
B (gardening—pleasant)	Planting beautiful flowers for 2 h
C (reading books—unpleasant)	Reading a driver’s manual to prepare for exam for 2 h
D (reading books—pleasant)	Reading a new novel by your favourite author for 2 h
Study design (between-subject):	
Separate evaluations	Joint evaluation
Schedule gardening tasks A and B; Schedule reading tasks C and D (random order)	Schedule all options together (A, B, C, D)
Dependent variable: diversification of options	
[’Imagine that on the next two Saturdays, you must plan when to do some reading and some gardening. On one Saturday, you will spend two tedious hours reading a driver’s manual in preparation for your licensing exam. On another Saturday, you will spend two pleasant hours reading a new novel by your favourite author. In addition to reading, on one Saturday, you will spend two boring hours weeding dandelions from your garden. On the other Saturday, you will spend two enjoyable hours planting flower bulbs. Choose which two tasks (one gardening, one reading) you would like to do for the first weekend, which means the two options not chosen will be done next weekend.’]	
Separate evaluations question set: (random order) — choose one gardening activity (for the first week) — choose one reading activity (for the first week) Joint evaluation question set: — choose one gardening activity and one reading activity (for the first week)	
Assumption check (extension): Need to ask out participants to rate how pleasant they find all four options. [‘Below are two reading tasks. Indicate which one you prefer doing:’] Question set 1: — Reading a new novel by your favourite author — Reading a driver's manual in preparation for a driver's exam Question set 2: — Weeding dandelions — Planting flower bulbs	

This study was an implicit assumption that participants consider reading a new novel by their favourite author as far more enjoyable than reading a driver’s manual for their driver’s exam and that they prefer planting flower bulbs over weeding dandelions. Though a reasonable assumption, we thought it relevant to ensure that participants indeed followed the target study’s assumptions. We therefore added a question explicitly asking participants for their preferences before they proceeded to the task, and only participants that had preferences in line with the target article’s assumptions were presented with that decision scenario.

### 4.4. Study 4 (Peanuts Effect)

Decision-makers faced with an uncertain gain option and a certain gain option typically tend to be risk-averse, yet the ‘Peanuts Effect’ is a phenomenon in which there is a reversal in smaller-valued risks. Meaning, that the smaller the stakes, the more likely decision-makers are to take the risk. It was mentioned in Read *et al*. [1] as one of the consequences of ‘narrow bracketing’ in which ‘repeated and seemingly inconsequential transactions can add up to significant total expenditures’. We therefore thought it relevant to examine if claimed consequences of bracketing are also replicable.

We reconstructed Study 4 as a test of the ‘Peanuts Effect’ based on a very brief description of the finding in the review article. Participants decided between a risky uncertain option, and a no-risk certain option. In the large-value condition, the numerical values of the options were 100 times bigger (10% chance to win $1000 or 100% gain of $100) than that in the low-value condition (10% chance to win $10 or 100% gain of $1). We summarized the study design in table 6. The findings were that people were more risk-averse in large-value bets compared with low-values bets.

**Table 6.** Study 4 (Peanuts Effect): study design (between-subject).

Small-value bets	Large-value bets
Choose either 10% chance to win $10 or 100% win of $1	Choose either 10% chance to win $1000 or 100% win of $100
Dependent variable: risk seeking versus risk-averse choice	
[‘Assume you are in a game show. The show host offered you the following two options. You must choose one of the two options. Which option will you choose?’]	

### 4.5. Study 5 (Determinants of Bracketing—cognitive inertia)

Study 5 involved making decisions on whether to take a risky bet with positive net value. Participants are told they will be facing that decision for five consecutive days. In the narrow bracketing scenario, they are told that they will make one bet each day, and then proceed to make the decision for the first day. In the broad bracketing scenario, they make the decisions for the five consecutive days together at the same time. We summarized the study design in table 7. The findings were that those in the broad bracketing condition were more likely to take the first bet than those in the narrow bracketing.

**Table 7.** Study 5 (cognitive inertia): study design (between-subject).

Separate betting (each day)	Joint betting (all 5 days together)
Betting decision for only the first day	Betting decision for 5 days at the same time
Dependent variable: taking the bet for first day	
‘Imagine that you are faced with 5 opportunities to make the following bet: 50% chance to win $40 and 50% chance to lose $25. The results will be revealed to you once a day for five consecutive days starting from today.’	
Separate: ‘Today, you only make the first bet.’, followed by a single bet for the first day.	
Joint: ‘You will have to make all 5 bets today.’, followed by 5 bets for each day: ‘Now please make your choice for the first/second/third/fourth/fifth day. On the first/second/third/fourth/fifth day, you will…’	
Option set: 1 = Take the bet—50% chance to win $40 and 50% chance to lose $25 0 = NOT take the bet	

### 4.6. Study 6 (Trade-offs across choices)

In Study 6, participants faced a decision of whether to spend money on a concert given different time frames and planned weekly spending. In the narrow bracketing scenario, the decision was framed in relation to a weekly budget cap, and the decision taking place at the end of a week with the weekly budget cap already met. In the broad bracketing scenario, the decision was framed in relation to a monthly budget cap, and the decision taking place at the end of the first week of the month. The findings were that people are more likely to spend money on a concert in the broad bracketing condition (monthly frame) compared with the narrow bracketing condition (weekly frame). We summarized the study design in table 8.

**Table 8.** Study 6 (trade-offs across purchase categories): study design (between-subject).

Week	Month	Extension: year
The budgeting period is a week	The budgeting period is a month	The budgeting period is a year
Scope is weekly	Scope is monthly	Scope is annual
Scenario: ‘Imagine that you are a poor university student, and you set aside [$50 per week / $200 per month /$2400 per year]. It’s the [last day of the week / seventh day of the month / seventh day of the year]. You have already spent $50 on entertainment. Tonight is a concert that you would like to attend, but the tickets cost $30. Will you purchase the $30 ticket and attend the concert?’		
Dependent variable: choice between the following two: ‘YES—I will purchase the $30 ticket and attend the concert’ ‘NO—I will NOT purchase the $30 ticket and will NOT attend the concert’		

Note. Prices were adjusted for inflation from approximately 1999 by doubling the mentioned prices. Source: CPI inflation calculator.

### 4.7. Study 7 (Pre-existing heuristics)

In Study 7, participants made decisions regarding booking desserts, which days out of a week to order a free dessert when they are away on a trip. In the weekly plan condition, participants ordered for all 7 days all at once on a single page. In the weekday–weekend condition, participants first ordered for weekdays, then for weekends on a separate page. We also added an extension condition, a single day condition, in which participants ordered for each day separately for the 7 days. We summarized the study design in table 9.

**Table 9.** Study 7 (pre-existing heuristics): study design (between-subject).

Weekly	Weekday–weekend	Extension: each day
Order dessert for the whole week together on 1 page	Order desserts for weekdays first, then separately for weekends	Seven separate decisions, one for each day, displayed in seven separate pages.
Dependent variable: number of days they will choose to eat dessert in a week		
‘Imagine that you are planning to attend an event in another city for a week (Monday to Sunday), and will need to eat all your meals in a hotel. They offer free bread pudding as dessert for every meal. The puddings are delicious but high in calories.’		
Whole week: ‘On which days of the week will you eat the bread pudding?’ [all on one page]		
Weekday–weekend: ‘You will first order your bread puddings for the weekdays on this page, and then in the next page will order puddings for the weekend.’ [one page for weekdays, one page for weekends]		
Each day: ‘In the following 7 pages, you will order your puddings for each day of the week, starting from Monday on this page, all the way to Sunday on the 7th page.’ [separate page for each day]		

Note. We adjusted ’a conference’ in the original scenario to ’an event’, to make it more generalizable and suitable for our target audience.

## 5. Extensions: Additional Bracketing conditions in Studies 6 and 7

According to the studies on bracketing effects as reviewed by Read *et al*. [1], the effect has mostly been described as a dichotomous manifestation, where choice sets are categorized as either ‘broad bracketing’ that leads to higher utility decisions or ‘narrow bracketing’ that leads to lower utility decisions in the experiments. Yet, we considered two ways of improving on this conceptualization and study design.

Firstly, dichotomization does not always closely mirror real-life conditions where choice sets are more complex than a simple binary classification. Take the example of the doughnut-eating decision mentioned earlier, apart from framing the question as ‘eating one doughnut after work today’ where every eating decision is made separately or ‘eating 260 doughnuts in a year’, where all the doughnut-eating decisions in a year are made all at once, it can also be framed as ‘eating five doughnuts a week’ or ‘eating 20 doughnuts a month’, where we bundle the doughnut-eating decisions in weeks or months. Examining many different categorizations and examining bracketing as a continuous measure may allow for a more nuanced understanding of the phenomenon and closer to real-life choices.

Therefore, we added two extensions, with an additional condition added to the experimental designs of Studies 6 and 7. In Study 6, we added a condition with broader bracketing (see table 8), and in Study 7, we added a condition with narrower bracketing (see table 9).

## 6. Pre-registration and open science

We provided all materials, data and code on: https://osf.io/vdqek/. This project received Stage 1 in-principle acceptance from Royal Society Open Science. We created a frozen pre-registration version of the entire Stage 1 packet (https://osf.io/9tz6h/) and proceeded to data collection. All measures, manipulations and exclusions conducted for this investigation are reported, and data collection was completed before analyses. This Registered Report was written using the Registered Report template by Feldman [33].

## 7. Method

### 7.1. Power and sensitivity analyses

We first aimed to calculate effect sizes (ES) and conduct a power analysis based on the effects reported in the target article, summarized in tables 2 and 10. Effect size, power and confidence intervals were all calculated with the help of a guide by Jané *et al*. [34] and R (v. 4.3.2 [35]), and then power analyses were conducted with R (v. 4.3.2 [35]) and GPower (v. 3.1 [36]) using data extrapolated from Li & Feldman [8]. The review article did not include many details about the descriptives, the statistical tests or effects, and so we could not calculate all effects, but of those we could Cohen’s *d* ranged from 0.38 to 1.28 and Cohen’s *w* from 0.18 to 0.88.

**Table 10.** Studies 2 and 7: summary of findings.

Summary of studies 2 and 7			
	***t* statistic**	***p***	**Cohen’s *d* [95% CI]**
Study 2—broad bracketing (*n* = 76)	1.65	0.099	0.38 [0.08, 0.83]
Study 2—narrow bracketing (*n* = 74)	5.50	<0.001	1.28 [0.77, 1.78]
Study 7 (*n* = 44)	4.00	<0.05	1.21 [0.56, 1.84]

Given the missing details, and the likelihood of the effects being an overestimation, we instead opted to conduct a power analysis based on an even weaker effect taken from a similar well-powered Replication Registered Report project that we conducted with Peer Community in Registered Reports (PCIRR) on diversification bias and partition dependence [8]. The effects we found in that project varied, and of those we chose the smallest effect: *d* = 0.27. Given the large sample and Registered Report process we thought it a fairly accurate and conservative estimate of effects in this literature. The phenomena seem very similar and related to each other, with similar designs and effects, both building on work by Read *et al*. with mentions of bracketing, partitioning and diversification. For example, Study 3 in Read *et al*. [1] investigates the notion of spreading out of favourable and unfavourable experiences under broad bracketing, which echoes the partitioning effect: the tendency to diversify when decisions are bundled together.

We concluded that the required sample size was 894 participants in total. This calculation is based on the effect size of *d* = 0.27, the smallest effect size mentioned in Li & Feldman [8] (95% power, alpha = 0.05). We provided more information in electronic supplementary material and accompanying code.

Our target sample is over three times larger than any of the of samples reported in the target article (Study 1: 236; Study 2: 112; Study 5: 143; Studies 3, 4, 6 and 7: not reported), meeting and exceeding the Simonsohn [37] small telescopes rule-of-thumb. A sensitivity analysis using Gpower [36] indicated that a sample of 894 would allow the detection of *w* = 0.13 for chi-squared goodness-of-fit test (95% power, alpha = 5%, one-tail), *d* = 0.27 (for contrasts in three condition with 298) to *d* = 0.22 (for contrasts in two conditions with 447 in each condition), and *f* = 0.13 for a 1 × 3 one-way ANOVA (95% power, alpha = 5%, one-tail).

### 7.2. Participants

We recruited a total of 896 Americans on Prolific (*M*_age_ = 42.63, s.d. = 13.69; 8 did not disclose their age; 406 females, 458 males, 32 other or did not disclose). We targeted Americans using Prolific’s filters. We restricted the location to the USA using ‘standard sample’, we set it to ‘Nationality: United States’, ‘Country of birth: United States’, ‘Minimum Approval Rate: 95, Maximum Approval Rate: 100’, ‘Minimum Submissions: 100, Maximum Submissions: 10 000’, ‘Place of most time spent before turning 18: United States’. We note that from the Stage 1 we updated ‘Minimum Submissions’ from 50 to 100, and ‘Maximum Submissions’ from 100 000 to 10 000 to target a slightly more experienced sample and adhere to Prolific’s new range restrictions. We also added the filter ‘Place of most time spent before turning 18: United States’ aiming to complement the ‘Nationality’ and ‘Country of birth’ filters aiming for participants born and raised in the United States. Our pay-off for the task was £1.20 per participant for an estimated 8 min survey (median time was 7:50 min), summarized by Prolific as a pay of £9.19 per hour (approx. US$12, as of 2024), exceeding targeted federal minimum wage of US$7.25 per hour.

### 7.3. Design: Replication and extension

All studies reviewed in Read *et al*. [1] were conducted separately with independent samples. We ran the seven studies together in a single unified data collection. The display of scenarios and conditions was counterbalanced using the randomizer ‘evenly present’ function in Qualtrics. Participants answered the seven decision scenarios from the seven studies in random order, and the conditions assigned in each study were also randomized. This unified design combining replications of several studies into a singular data collection was previously tested successfully in many of the replications and extensions conducted by our team (e.g. [13,38–40]), and is especially powerful in addressing concerns about the target sample (e.g. naivety and attentiveness) when some studies replicate successfully whereas others do not, as well as in allowing for drawing inferences about links between the different studies and consistency in participants’ responding to similar decision-making paradigms.

We summarized the experimental designs of the seven studies in tables 3–9, and our adjustments to the target article are in table 11.

**Table 11.** Adjustments to the target article’s methods and design.

Study	Factor	Target article	Adjustment in current study	Reason for change/justifications
1	Procedure	Two separate studies on bracketing and outcome editing	Combined two investigations into one unified investigation	Minimize need for adjustments in data collection methods and shorten questionnaire length
2	Dependent variable	Scale from $20 000 and $40 000 (points unspecified in target article)	11-point scale from below $50 000 to above $150 000	Modified the salary range to adjusting for inflation and reflecting salary standards in 2024
3	Assumption check	No testing of the preference assumptions	Added two checks	Verify that the participants’ preference aligns with the target’s assumptions
3	Wording	Pennsylvania driver’s manual	A driver’s manual	Modified to increase generalizability
6	Prices	Weekly budget: $25/week, $100/month	Weekly budget: $50/week, $200/month	Modified the prices adjusting for inflation
7	Study design	Prompt: ‘Imagine you are planning to attend a conference in Philadelphia’	Prompt: ‘Imagine you are planning to attend a conference in another city’	Modified to our target sample across the USA and to increase generalizability

## 8. Procedure

We reconstructed the target’s stimuli and adjusted it to an online Qualtrics survey based on the information provided in the article.

Participants indicated their consent, with four questions confirming their eligibility, understanding and agreement with study terms, which they must answer with a ‘yes’ and required responses in order to proceed to the study. Three of the four questions also served as attention checks, with the order of the options being rotated (yes, no, not sure).

The question order and conditions assigned for each study in this unified data collection were randomized using the ‘randomizer’ and ‘evenly present’ functions. In Study 3, we used assumption checks to ensure that participants fit the criteria for participating. This is a deviation from the target’s procedure and was meant to ensure that participants fit the prerequisites for the study.

At the end of the experiment, participants answered a number of funnelling and demographic questions and were debriefed.

## 9. Study designs and measures

All studies were using a between-subjects design. Participants were randomly assigned to one of the two or three conditions within each study. We provided a detailed description of the studies in the introduction and summarized the manipulations, experimental designs and measures in tables 3–9.

### 9.1. Assumption checks (extension)

Study 3 investigates the tendency to spread out ‘desirable’ and ‘undesirable’ experiences. To ensure participants perceive our ‘desirable’ options as such and vice versa, we added an assumption check at the end of the survey used to ensure participants did not perceive our ‘desirable’ options as ‘undesirable’. We note that this is a needed deviation from the target article that provides for a more conservative test than the target’s design.

This check included two questions where participants were asked if they prefer ‘Reading a new novel by your favourite author’ to ‘Reading a driver’s manual in preparation for a driver’s exam’ and ‘Weeding dandelions’ to ‘Planting flower bulbs’. Participants who prefer ‘Reading a driver’s manual’ to ‘Reading a new novel by your favourite author’ and/or ‘Weeding dandelions’ to ‘Planting flower bulbs’ were excluded from this study.^1^

## 10. Adjustments and deviations

We made several adjustments to the original study. These changes were made to facilitate participants’ understanding and immersion, and are all summarized in table 11.

### 10.1. Evaluation criteria for replication findings

We aimed to compare the replication effects with the original effects (as shown in tables 3, 10 and 12 using the criteria set by LeBel *et al*. [41].

**Table 12.** Studies 1, 5 and 6: summary of findings.

Summary of Studies 1, 5 and 6				
	***X*** *^2^*	**d.f.**	***p***	**Cohen’s *w* and 95% CIs**
Study 1—Option A/B (*n* = 150)	15.29	1	<0.001	0.29 [0.16,0.43]
Study 1—Option C/D (*n* = 150)	150.46	1	<0.001	0.88 [0.74,1.00]
Study 5 (*n* = 143)	4.57	1	<0.05	0.18 [0.00, 0.35]
Study 6	7.7	1	<0.005	N/A

Note. Target article’s Study 6 did not report the number of participants, and Studies 1 (2.2) and 4 did not provide sufficient details to calculate effects.

We pre-registered our overall strategy to conclude a successful replication if 5 to 7 out of 7 showed a signal in the same direction as the target article, a failed replication if 0 to 2 out of 7, and mixed findings if 3 to 4 successful out of 7. In the case of Study 1 combining two designs, we planned to conclude a successful replication if both sections were supported.

### 10.2. Replication closeness evaluation

We provided details on the classification of the replications using the criteria by LeBel *et al*. [42] criteria in table 13 (see section ‘replication closeness evaluation’ in electronic supplementary material). All studies were evaluated with these criteria except for Study 4, as no actual data collection was reported in the target article. We summarized the replication of all other studies as a ‘close’ replication.

**Table 13.** Classification of the replication based on LeBel *et al*. [42].

Design facet	Replication	Details of deviation and severity
Effect/hypothesis	Same	
IV construct	Same	
DV construct	Same	
IV operationalization	Same	
DV operationalization	Same	
IV stimuli	Similar	
DV stimuli	Similar	Study 2 salaries adjusted to fit inflation Study 6 prices adjusted to fit inflation
Procedural details	Similar	Assumption check was added for Study 3
Physical settings	Different	Our study was conducted online using Qualtrics and participants recruited using Prolific. We have no details about the studies reviewed, yet given it was prior to 1999, we assume it was different.
Contextual variables	Different	Our study was conducted in 2024. The original studies were conducted on or before 1999. We do not have much information about the context of those studies.
Population (e.g. age)	Different	Many of the studies were conducted on undergraduates, whereas our sample was a more diverse population on Prolific.
Replication classification	Close replication	

Note. Criteria for evaluation of replications by LeBel *et al*. [42]. The ‘Similar’ category was added to the LeBel *et al*. [42] typology to refer to minor deviations or extensions aimed at adjusting the study to the target sample that are not expected to have major implications on replication success. Study 4 is excluded from this analysis, given that no details were reported for this study in the target article.

## 11. Data analysis strategy

We aimed to replicate the analytical methods reported in Read *et al*. [1]. We also referenced the originators of the reviewed studies for more information regarding results and study design, namely Tversky & Kahneman [22] for Study 1 and Hsee [15] for Study 2.

Our analyses were conducted with R (v. 4.3.2) using packages ‘tidyverse’ [43], ‘jmv’(v. 2.4.11) , ‘ggstatsplot’ [44], ‘ufs’ [45].

### 11.1. Order effects

One deviation from the target article is that all participants completed all scenarios in random order. We considered this to be a stronger design with many advantages, yet one disadvantage is that answers to one scenario may bias participants’ answers to the following scenarios.

We therefore pre-registered that if we failed to find support for the core hypotheses of the target article that we rerun exploratory analyses for the failed study by focusing on the participants that completed that study first, and examine order as a moderator. To compensate for multiple comparisons and increased likelihood of capitalizing on chance, we set the alpha for the additional analyses to a stricter 0.005.

### 11.2. Outliers and exclusions

We did not classify any cases as outliers. We included all the data collected in our analysis for those who successfully completed the entire study.

### 11.3. Bayesian analyses

We pre-registered that in case we failed to find support for the hypothesis for any of the studies, then we would run a complementary Bayesian analysis for that study using a prior of 0.707 to quantify support for the null, and those were added using the ggstatsplot figures provided for each study.

## 12. Results

**Figure 1.**
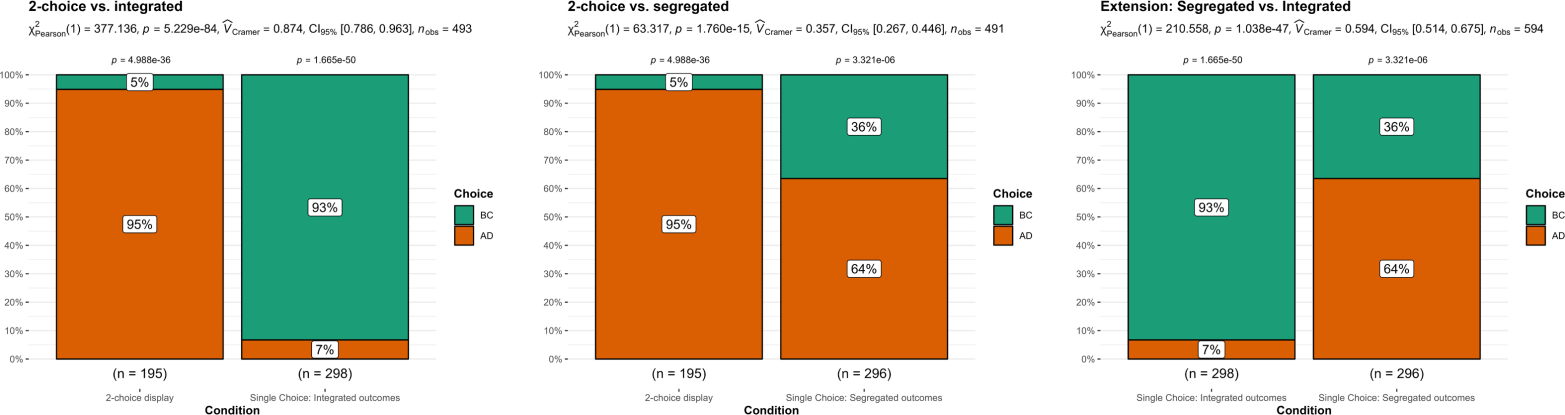
Study 1: comparison of choice.

**Figure 2.**
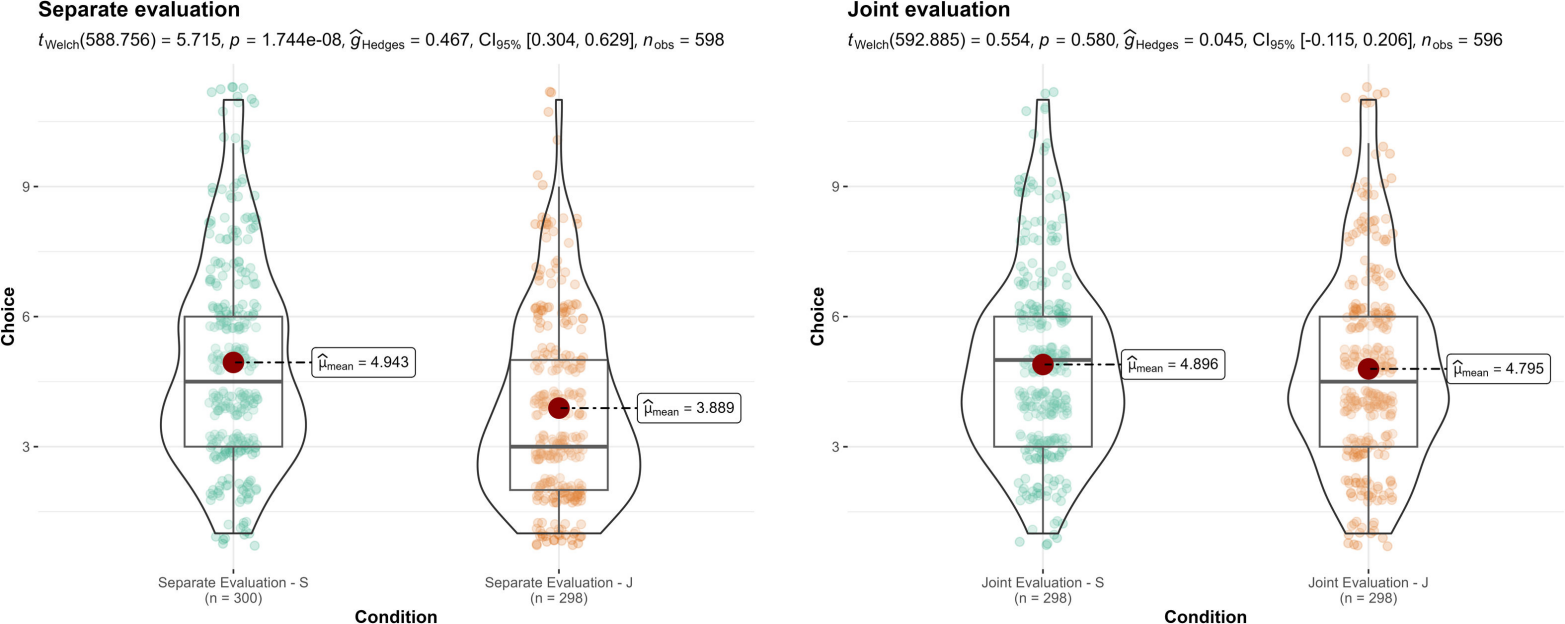
Study 2: comparison of salary evaluation of candidates J and S in joint and separate evaluation mode.

**Figure 3.**
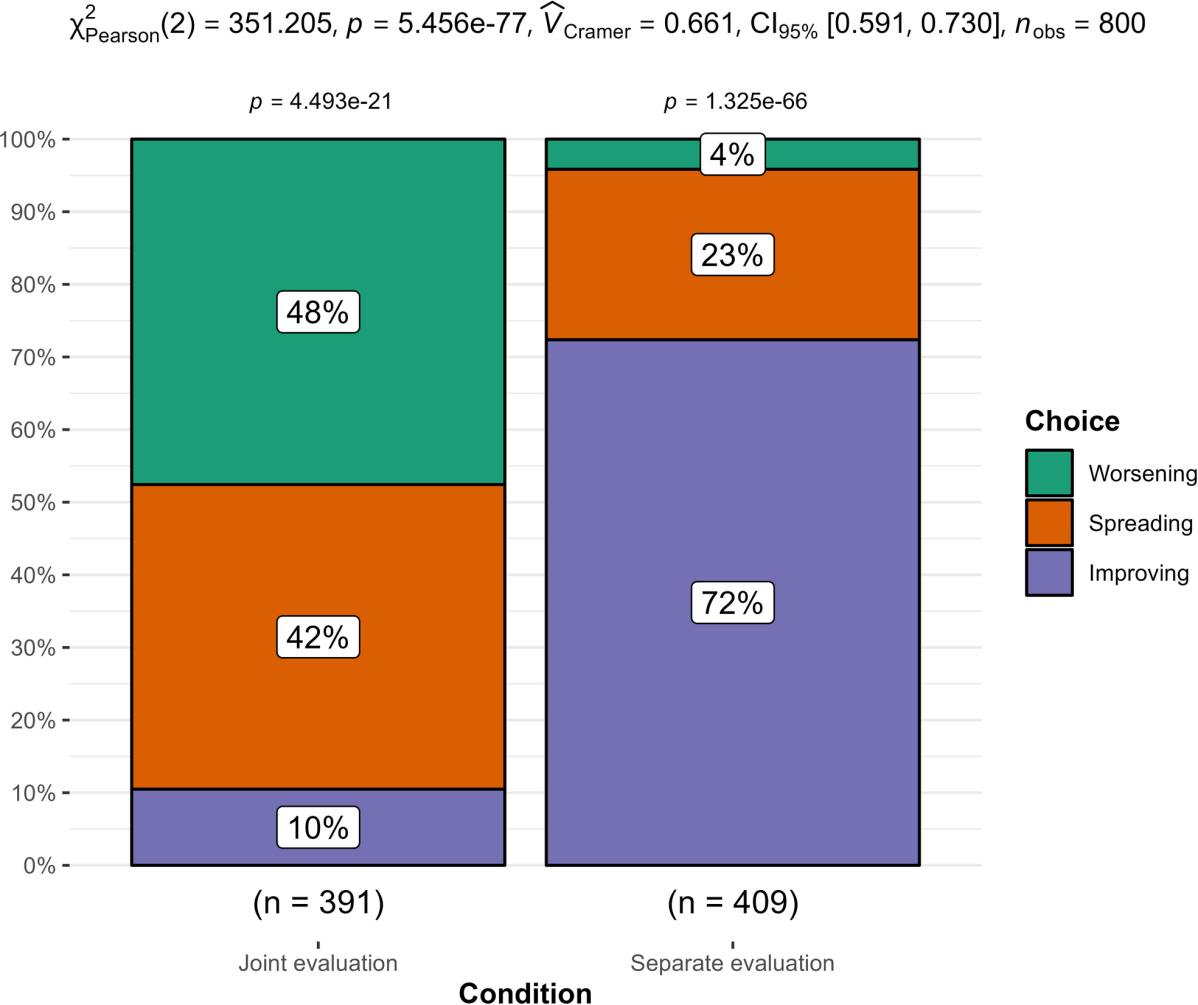
Study 3: comparison of activity sequence chosen between separate and joint evaluation mode.

**Figure 4.**
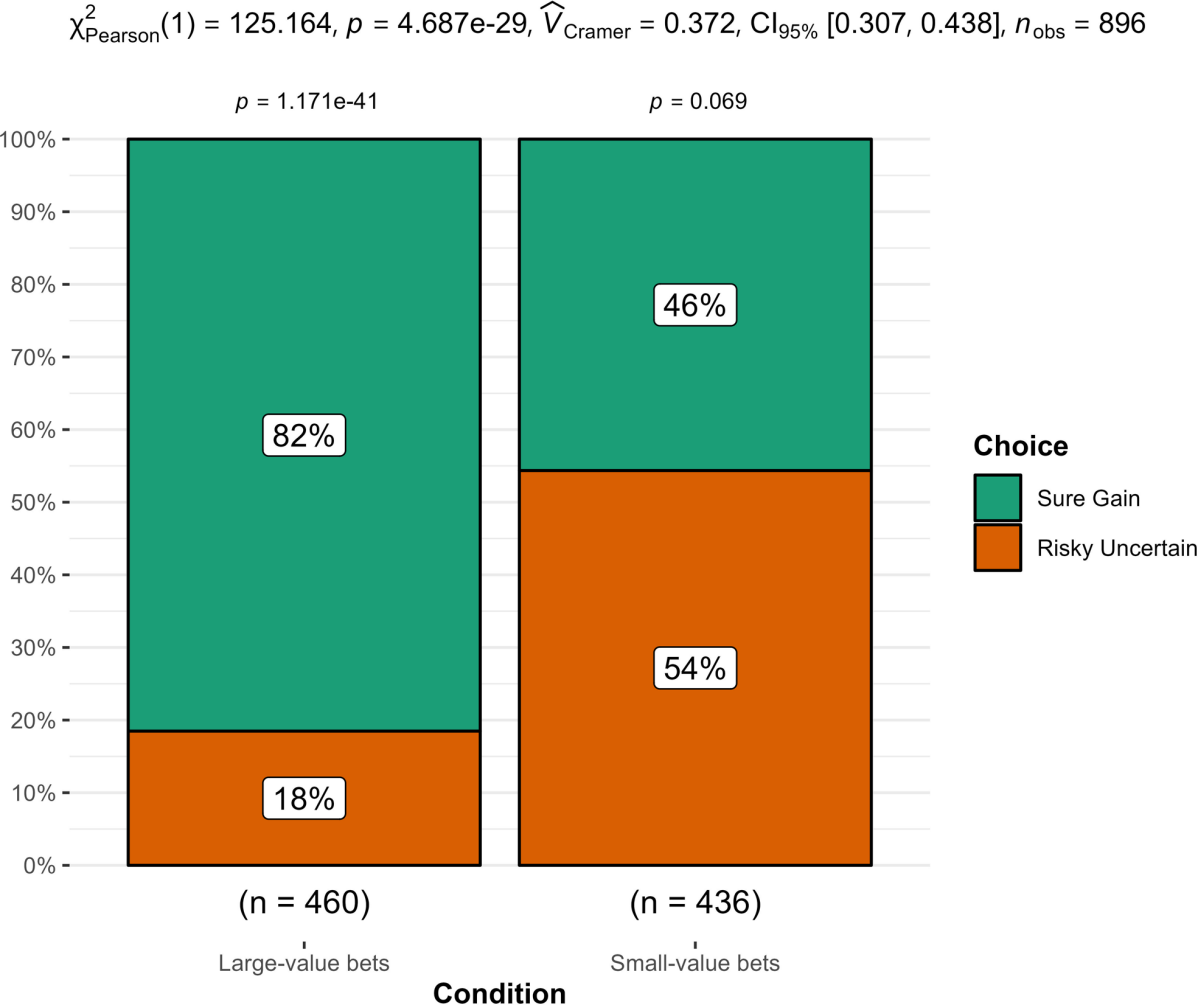
Study 4: comparison of choice contrasting small versus large-value choice sets.

**Figure 5.**
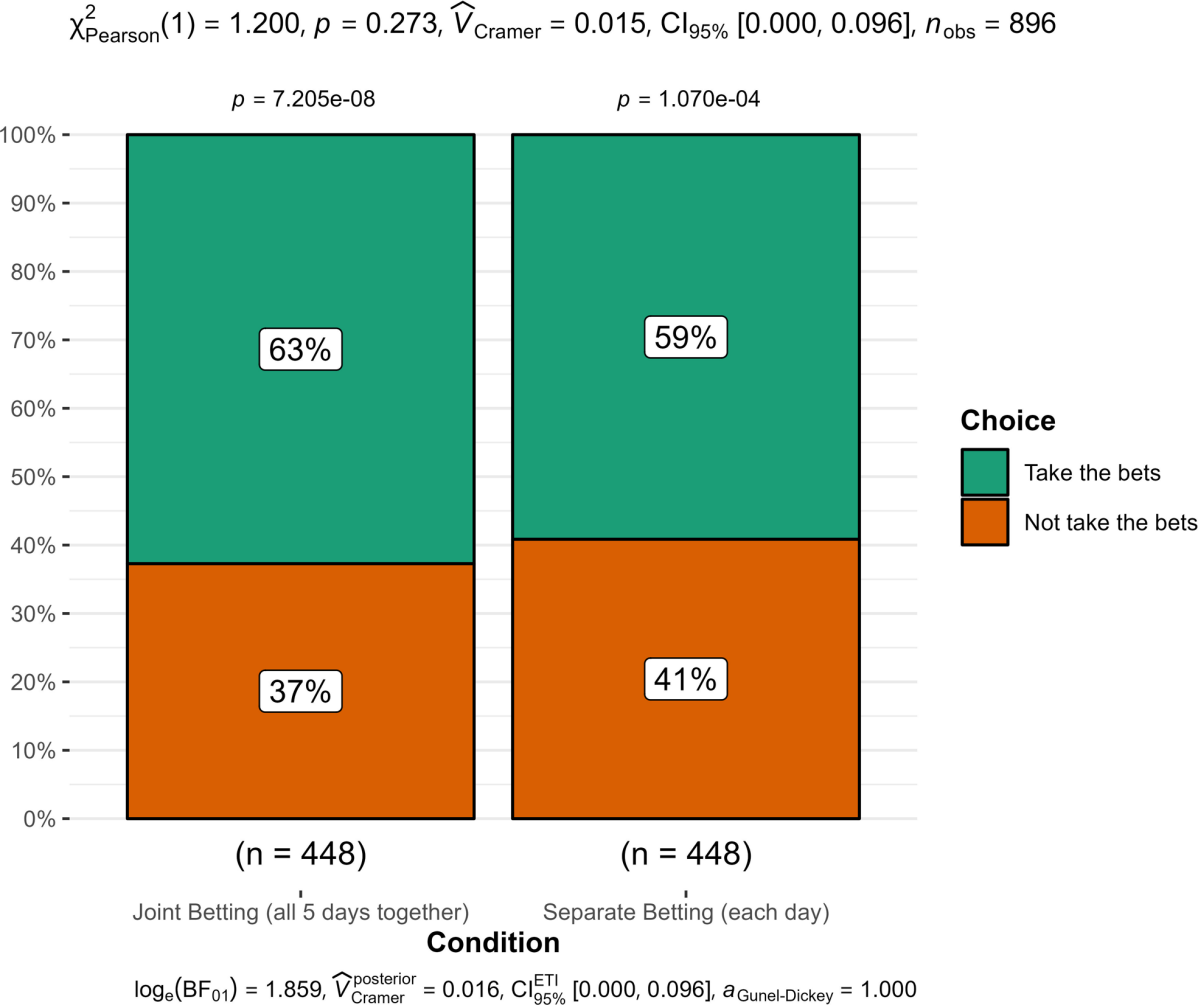
Study 5: comparison of bet-taking choices between joint and separate betting conditions.

**Figure 6.**
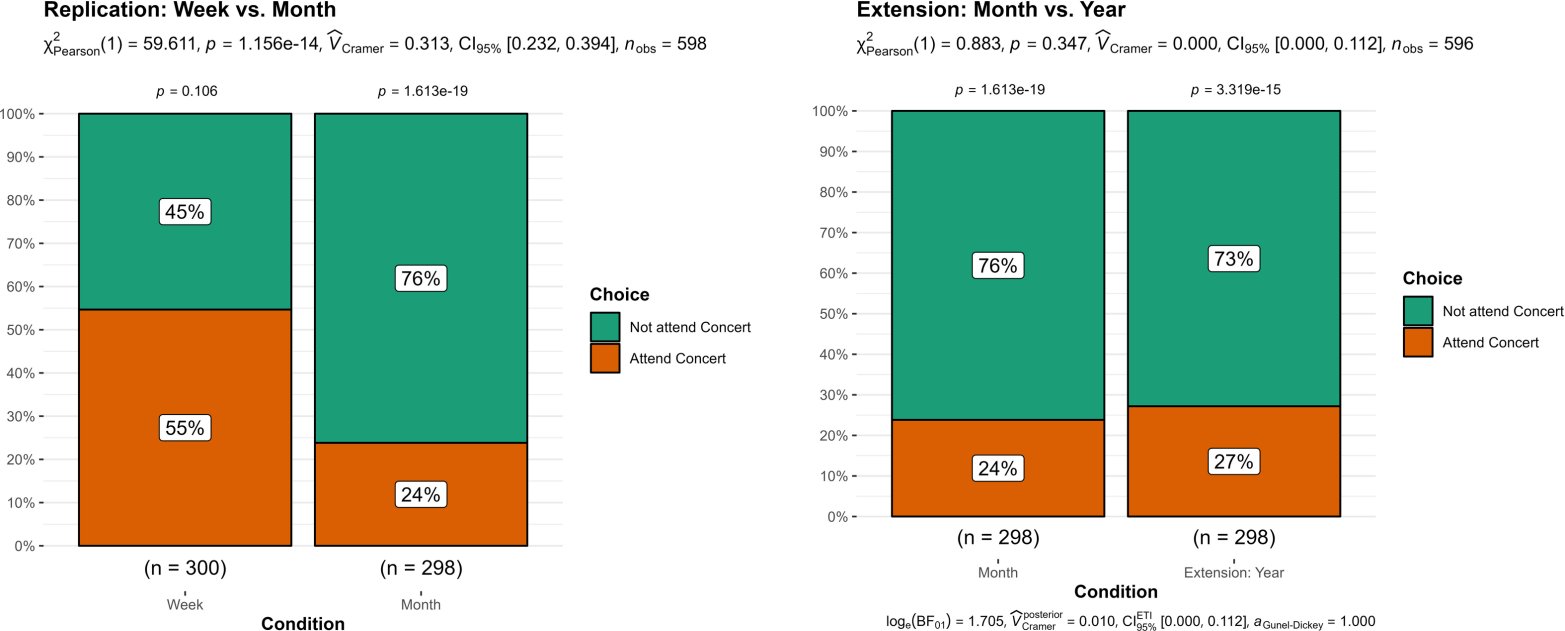
Study 6: concert-going decision contrasting scope of week versus month (replication) and month versus year (extension).

**Figure 7.**
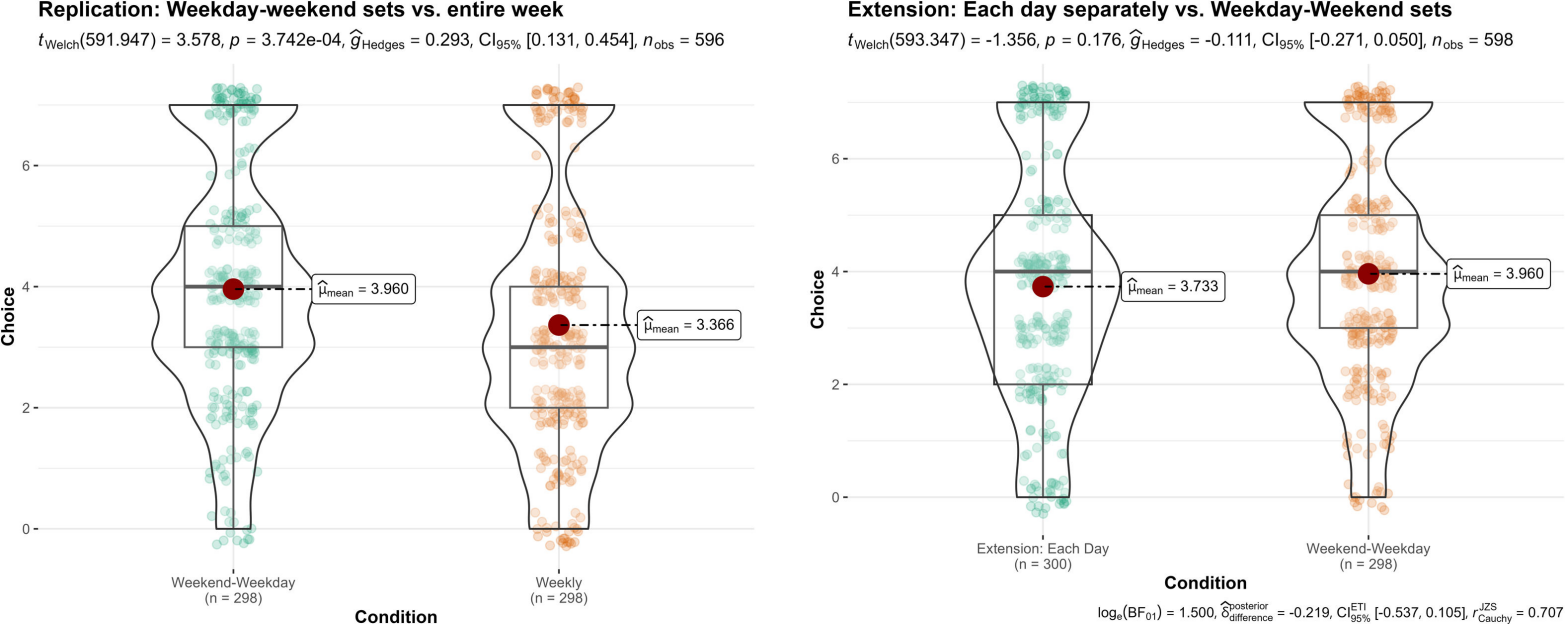
Study 7: comparison of aggregated pudding-eating decisions between weekly, weekly+weekend and daily presentation of choice set.

We summarized the descriptives in table 14 and statistical tests in tables 15 and 16 with plots in figures 1–7. We created the plots using the ggstatsplot R package [44].

**Table 14.** All studies: descriptives.

Study	Dependent variable	Conditions		
1		Single choice: integrated outcomes (*n* = 298)	Two-choice display (*n* = 302)	Single choice: segregated outcomes (*n* = 296)
	AC	N/A	74 (24.5%)	N/A
	AD	20 (6.7%)	185 (61.3%)	188 (63.1%)
	BC	278 (93.2%)	10 (3.3%)	108 (36.2%)
	BD	N/A	33 (10.9%)	N/A
2		Separate evaluation (*n* = 598)	Joint evaluation (*n* = 298)	
	Evaluation for J	3.89 [2.12] (*n* = 298)	4.80 [2.27] (*n* = 298)	
	Evaluation for S	4.94 [2.38] (*n* = 300)	4.90 [2.17] (*n* = 298)	
3		Joint evaluation (*n* = 391)	Separate evaluation (*n* = 409)	Excluded (*n* = 96)
	Improving sequence	41 (10.5%)	296 (72.4%)	N/A
	Spreading out sequence	164 (41.9%)	96 (23.5%)	N/A
	Worsening sequence	186 (47.6%)	17 (4.1%)	N/A
4		Large-value bets (*n* = 460)	Small-value bets (*n* = 436)	Total (*n* = 896)
	Risky bet	377 (81.9%)	200 (45.8%)	577 (64.4.3%)
	Sure-gain bet	83 (18.0%)	236 (54.1%)	318 (35.6%)
5		Joint betting (all 5 days together) (*n* = 448)	Separate betting (each day) (*n* = 448)	Total (*n* = 896)
	Take the bet	282 (36.8%)	264 (58.9%)	546 (60.9%)
	Not take the bet	166 (37.1%)	184 (41.1%)	350 (39.1%)
6		Week (*n* = 300)	Month (*n* = 298)	Extension: Year (*n* = 298)
	Attend concert	135 (45.0%)	226 (75.8%)	218 (73.2%)
	Not attend concert	165 (55.0%)	72 (24.2%)	80 (26.8%)
7		Extension: Each day (*n* = 300)	Weekend–weekday (*n* = 298)	Weekly (*n* = 298)
	Days eating dessert	4.13 [1.82]	4.15 [1.80]	3.66 [1.91]

Note. The numbers denote counts of participants selecting each option, percentages are shown in parentheses. ‘*n’* indicates sample size for that condition. For Studies 2 and 7, the data is presented as MM.MM [SD.SD], where MM.MM indicates mean and SD.SD indicates standard deviation. *n* indicates sample size for that condition.

**Table 15.** Studies 1, 3, 4, 5, 6: summary of findings.

Replication findings							Original article findings				
**Study**	**Conditions**	**d.f.**	***X^2^***	***p***	**Cramer’s *V***	**95% CI**	***X^2^***	***p***	**Cramer’s *V***	**95% CI**	**Interpretation**
1	Two-choice display versus single choice: segregated outcomes	1	63.32	<0.001	0.36	[0.28, 0.43]	N/A	N/A	N/A	N/A	Signal, same direction
1	Two-choice display versus single choice: integrated	1	377.14	<0.001	0.87	[0.85, 0.89]	−15.29 150.46	<.001 <.001	−0.25 0.84	[−0.38, −0.13] [0.71, 0.97]	Signal, same direction
1	Extension: single choice: integrated versus segregated outcomes	1	210.56	<0.001	0.59	[0.54, 0.65]	—	—	—	—	Signal
3	Separate versus joint evaluation modes	2	351.20	<0.001	0.66	[0.62, 0.70]	—	—	—	—	Signal, same direction
4	Large versus small-valued bets	1	125.16	<0.001	0.37	[0.32, 0.43]	—	—	—	—	Signal, same direction
5	Separate versus joint betting	1	1.20	0.273	0.02	[0.00, 0.10]	4.57	<.05	0.18	[0.00, 0.35]	No signal—inconsistent
6	Week versus month	1	59.61	<0.001	0.31	[0.25, 1.00]	7.7	<.005	N/A	N/A	Signal, same direction
6	Extension: month versus year	1	0.88	0.347	0.00	[0.00, 1.00]	—	—	—	—	No signal

Note. N/A = not available, not reported in the target article

**Table 16.** Studies 2 and 7: summary of findings.

		Replication findings					Original article findings				
**Study**	**Conditions**	**d.f.**	***t***	***p***	**Cohen’s *d***	**95% CI**	***t***	***p***	**Cohen’s *d***	**95% CI**	**Interpretation**
2	Separate evaluation	598	5.72	<0.001	.46	[0.30, 0.63]	5.50	<0.001	1.28	[0.77, 1.78]	Signal, inconsistent, weaker
2	Joint evaluation	596	0.55	0.58	0.05	[−0.12, 0.21]	1.65	0.099	−0.38	[−0.83, 0.08]	No signal— consistent
2	Comparison between separate and joint evaluation modes				Separate > joint, no direction reversal				Separate > joint, direction reversal		Consistent, without reversal
7	Whole week versus weekday–weekend	596	3.58	<0.001	0.29	[0.13, 0.45]	4.00	<0.05	1.21	[0.56, 1.84]	Signal – consistent
7	Extension: weekday–weekend versus each day	598	−1.36	0.17	0.11	[−0.27, 0.05]	—	—	—	—	No signal

### 12.1. Study 1 (Choice Bracketing and outcome editing)

We summarized the findings in figure 1. We conducted two chi-square tests of independence on choice contrasting two-choice display versus single-choice segregated and then two-choice display versus single-choice integrated. For these analyses, to allow for a comparison of the two conditions, we excluded participants who chose ‘AC’ and/or ‘BD’ in the two-choice display scenario to only compare the proportions of individuals making ‘high-utility’ decisions (i.e. BC) and ‘low-utility’ decisions (i.e. AD) under the two conditions.

Mirroring the target’s ‘choice bracketing’ section, we contrasted two-choice display versus single-choice integrated and found support for a main effect of bracketing on the choice of bets (*X^2^* (1, *n* = 493) = 377.14, *p* < 0.001; *V* = 0.87, 95% CI [0.85, 0.89]). Specifically, 93% of participants made high-utility decisions (i.e. BC) in the single-choice integrated condition, compared with 5% in two-choice display condition.

Mirroring the target’s ‘outcome editing’ section, we contrasted two-choice display versus single-choice segregated and found support for a main effect of bracketing on the choice of bets (*X^2^* (1, *n* = 491) = 63.32, *p* < 0.001; *V* = 0.36, 95% CI [0.28, 0.43]). Specifically, 36% of participants made high-utility decisions in the single-choice segregated condition, compared with 5% in two-choice display condition.

We also conducted an additional extension to investigate the effect of choice set presentation on decision-making. We conducted a chi-square test of independence contrasting single-choice integrated with the single-choice segregated conditions. We found support for a main effect on the choice of bets (*X^2^* (1, *n* = 594) = 210.56, *p* < o.001; *V* = o.59, 95% CI [0.54, 0.65]). A total of 93% of participants made high-utility decisions in the single-choice integrated condition, compared with 36% in the single-choice segregated condition.

### 12.2. Study 2 (Joint versus separate evaluation of alternatives; Hsee, [15])

We summarized the findings in figure 2. We conducted a Welch independent *t*‐test contrasting candidates J and S in separate and joint evaluations and found support for differences in the separate evaluation mode (*t*(588.8) = 5.72, *p* < 0.001; *g* = 0.47, 95% CI [0.30, 0.63]) but not in the joint evaluation mode (*t*(592.9) = 0.55, *p* = 0.580; *g* = 0.05, 95% CI [−0.12, 0.21]). Although we did not observe a complete reversal as in the target article, the reversal in the target article was not significant to begin with, and the confidence intervals of the effects of candidates J and S did not overlap,

suggesting a weaker different effect for joint evaluations than for separate evaluations.

### 12.3. Study 3 (Scheduling future experiences)

We summarized the findings in figure 3. We computed the dependent variable such that the planning sequence of choosing both ‘unpleasant’ events for the first week, and both ‘pleasant’ events for the second week was classified as ‘improving’, the sequence of choosing one ‘pleasant’ and one ‘unpleasant’ for each week was classified as ‘spreading’, and the sequence of choosing both ‘pleasant’ activities for the first week and ‘unpleasant’ activities for the second week was classified as ‘worsening’.

We included a manipulation check and based on the pre-registered criteria filtered out those who perceived ‘weeding’ and ‘reading a driver’s manual’ as more preferable than ‘planting flower bulbs’ and ‘reading a novel by your favourite author’ as they did not fit the criteria for the study where negative emotional evaluations are associated with the former (i.e. weeding and reading the manual), and vice versa. Overall, 96 participants failed the assumption tests, 58 in the joint evaluation condition and 38 in the separate evaluation condition.

We conducted a chi-square test of independence between separate evaluations and joint evaluations conditions and found support for a main effect (*X^2^* (2, *n* = 800) = 351.20, *p* < 0.001; *V* = 0.66, 95% CI [0.62, 0.70]). Specifically, 42% of participants chose a ‘spreading’ sequence in the joint evaluation mode condition, compared with 23% in the separate evaluation mode condition.

Additionally, we observed a reversal for worsening and improving. Whereas 48% of participants chose a worsening sequence in the joint evaluation mode, only 4% chose that sequence in the separate evaluation mode. Conversely, only 10% of participants in the joint evaluation condition chose an improving sequence, whereas 72% of participants chose it in the separate evaluation condition.

### 12.4. Study 4 (Peanuts Effect)

We summarized the findings in figure 4. We conducted a chi-square test of independence contrasting the small-valued bet and the large-valued bet and found support for a main effect (*X^2^* (1, *n* = 896) = 125.16, *p* < 0.001; *V* = 0.37, 95% CI [0.32, 0.43]). Specifically, 82% of participants chose the ‘sure gain’ option in the large-value bets conditions, compared with 46% in the small-value bets condition.

### 12.5. Study 5 (Determinants of bracketing—cognitive inertia)

We summarized the findings in figure 5. We conducted a chi-squared test of independence comparing joint betting (all 5 days together) with separate betting (each day) and found no support for a main effect of mode on choice (*X^2^* (1, *n* = 896) = 1.20, *p* = 0.273; *V* = 0.02, 95% CI [0.00, 0.10]).

Following the pre-registration plan in case of a failure to find support for the target’s findings, we ran an additional analysis only on the participants that answered this study first, with similar results and still no support (*X^2^* (1, *n* = 131) = 1.80, *p* = 0.180; *V* = 0.08, 95% CI [0.00, 0.28]).

### 12.6. Study 6 (Trade-offs across choices)

We summarized the findings in figure 6. We conducted a chi-squared test and found support for differences between the week and month conditions (replication: *X^2^* (1, *n* = 598) = 59.61, *p* < 0.001; *V* = 0.32, 95% CI [0.25, 1.00]). Specifically, 45% of participants chose to attend the concert in the week condition, compared with 76% in the month condition.

We also conducted an additional extension to investigate the effect of enlarging the scope. A chi-squared test of independence found no support for a main effect for differences between month and year (extension: *X^2^* (1, *n* = 596) = 0.88, *p* = .347; *V* = 0.04, 95% CI [0.00, 0.12]).

### 12.7. Study 7 (Pre-existing heuristics)

We summarized the findings in figure 7. We conducted a Welch independent samples *t*‐test and found support for differences between the accumulated dessert-eating days in weekly versus weekday–weekend conditions (replication; *t*(591.9) = 3.58, *p* < 0.001; *g* = 0.29, 95% CI [0.13, 0.45]), with participants more likely to eat more dessert in the weekday–weekend condition (*M* = 3.96, s.d. = 1.97) than in the weekly condition (*M* = 3.37, s.d. = 2.09).

We found no support for our extension on the differences between weekday–weekend versus each day (extension; *t*(593.3)= −1.36, *p* = 0.176; *g* = −0.11, 95% CI[−0.27, 0.05]). We ran an additional order effect analysis examining only participants who answered this study first and found similar results (*t*(86.15) = 0.17, *p* = 0.864; *g* = 0.04, 95% CI [−0.38, 0.45]).

## 13. Discussion

We conducted a close replication of seven studies reviewed by Read *et al*. [1]. Overall, the replication findings are mostly consistent with the target article. In addition, we conducted several extensions manipulating scope and found no indication for scope as a continuous range. We summarized our findings and a comparison with the findings reported in the target article in tables 15 and 16.

### 13.1. Studies 1 and 4—Risk taking

We successfully replicated Study 1. The findings reveal a striking effect showing that different presentation formats greatly influence decision-making processes.

The unusually large effects (i.e. Cramer’s *V* values of 0.87 and 0.59) underscore the susceptibility of individuals in the decision-making process to subtle presentational alterations of choice options. These effects position bracketing as possibly one of the strongest interventions within the field of decision-making, and we see great potential in extending these findings to other domains and testing its applications more widely.

In Study 4, we found support for the Peanuts Effect, showing a larger tendency for risk seeking in low-value bets compared with high-value bets [46]. The mention of Peanuts Effect was probably meant more as an illustration rather than a description of an experiment, yet we thought it relevant to try and test the described intuition. Future research can build on that to better position the Peanuts Effects in the context of bracketing, possibly by presenting one large bet versus a series of much smaller bets that end up amounting to the same value, either separately (as in the replication of Study 5) or together.

### 13.2. Studies 2 and 3—Joint versus separate evaluation modes

Study 2 replicated successfully, with weaker effects. The target article documented a complete preference reversal when comparing joint and separate evaluations, and in our replication we did not observe that reversal, but rather that the joint evaluation mode had a weaker null effect compared with the separate evaluation condition.

One potential explanation reflecting a limitation that was discussed in the peer review process is regarding the Grade Point Average (GPA) information. The American education system amended its GPA range from a 5-point to a 4-point scale, yet we decided to keep the original 5-point description of the GPA range instead of converting it to a 4-point scale, to stay closer to the target’s stimuli. We made that decision because we are unsure of the potential impact of such a change. We tried to address that limitation by emphasizing that the GPA is presented on a ‘5-point scale’ in our question prompt across all conditions. Hence, future research may address this by reverting to a 4.0 GPA scale to eliminate the possibility of comprehension error and more accurately study the effect of joint evaluation on the salary evaluation of the participants.

The more likely possibility is simply the shift in preferences regarding those candidates in our context, given the many changes since the twentieth century, in the passing of time, the context, the sample and how those may have affected the evaluation of such candidates and their skills. Given the overall successful pattern, we see no need to overthink these differences.

In Study 3, we also saw a successful replication, and, as expected, joint evaluation mode led to more spreading of desirable and undesirable experiences over time.

What remains unclear is which of the two modes leads to the more optimal choice. The main argument was that joint evaluation mode helps decision-makers evaluate all options together and better align decisions with goals to help them better optimize hiring decisions and their emotional utility. However, this depends on one’s goals, and so especially in the case of hiring in Study 2, it might be possible that seeing all options together creates a new reference point and a comparison that shifts the decision-maker further away from the optimal point. Therefore, in future studies, rather than paternalistically assuming which of the two results is the higher utility option, it would be best to incorporate some evaluation of the decision-maker’s own goals and compare those with both evaluation modes to see which ones gets the decision-maker closer to their goals.

### 13.3. Studies 6 and 7—Planning and time

The findings of our replication of Studies 6 and 7 also closely mirror the ones reported in the target article. Yet, in our construction of the extensions and examining the results, we see a gap with the link between Study 6 and bracketing, and consider this study and its evidence as being a demonstration of mental accounting ([11]; see recent replication by Li & Feldman [8] and goals as reference points ([47]; see recent replication by Au & Feldman [7]), possibly as an instance of bracketing. In this experiment, it was not so clear whether the experiment was about a narrow focus on the week, or rather whether one is calculating against the reference point determined by the fiscal period and is considering the limit previously set for the mental account. Our Study 6 extension, where we failed to observe differences between month and year, suggests that this was less about the fiscal period and more about the account. Future research could try and better formulate the connection between bracketing, mental accounting, and goals as reference points, to try and disentangle these concepts and design cleaner experimental designs pivoting those against each other.

In our extensions in both Study 6 and Study 7, we tried to examine whether bracketing should be considered dichotomous, in which case we need clear falsifiable criteria regarding how to set that dichotomy, or whether bracketing should be considered as a continuous variable. Future research should build on that attempt to both better define what bracketing is, how it is defined, set clear testable hypotheses and then determine whether this should be thought of as continuous.

### 13.4. Study 5

In our replication of Study 5, we found no support for the original findings. This failure may be due to our inability to construct a faithful translation of the replication to survey format. The original study took place in a classroom, where ‘taking all five bets at once’ and ‘taking one bet a day for 5 days’ was realistic given that the participants returned to the classroom in consecutive weeks and were expecting to continue the study. It is likely that in our one-off survey the set-up was not realistic enough, and so participants knew that they were only making the decision once, and so this affected their responses to the initial bet. Future studies may further investigate the effect of cognitive inertia by amending the study design from a one-off survey to a multiple time-points multiple bets to provide a more faithful replication.

## 14. Defining Bracketing

We focused on the practical replicability of studies from a review article said to be about bracketing. Yet, in our peer review and through the process, we felt that as a community we need to discuss and address major challenges regarding the definition and the conceptual clarity of ‘choice bracketing’. In the field of judgement and decision-making literature, constructs sometimes suffer from vague and ambiguous definitions, which translates to underspecified and lacking theory that hinders falsifiability and consistency. Read and colleagues [1] defined *choice bracketing* as ‘designating groups of individual choices into sets’, yet this definition can be said to coincide with Goffman’s [48] notion of ‘framing’ and ‘reframing’, which describes ‘looking at a single choice through the frame of many choices’. In our discussion of Study 6, we pointed out similar overlap with other ideas of reference points and mental accounting. Similarly, in our peer review discussions we also often wondered whether bracketing is simply a name for joint versus separate evaluation modes, represents a broader phenomenon, or perhaps a different phenomenon altogether. We did not consider it our role as replicators to solve these major issues in the literature but rather to focus on whether the empirical evidence claimed to represent bracketing is replicable and solid. However, we agree with our reviewers that for bracketing to have academic and practical impact, and to live up to its full potential, researchers must go back to the foundations, rethink, organize and tackle these challenges.

As a first step, bracketing, framing, reference points and mental accounting need a broad scholarly discussion that would result in better theoretical clarity and definitions. For example, some might argue that the concept of choice bracketing can simply be interpreted as a subclass of framing instead of a stand-alone construct, as it focuses on the mental evaluation of choice sets through adjusting the contextual background. Some studies emphasize the behavioural aspects of bracketing, focusing on the observable differences in decision-making, whereas others focus on the cognitive process underlying bracketing as they examine how individuals mentally evaluate and organize choices.

Within the bracketing construct, the definitions of ‘broad’ and ‘narrow’ bracketing are also not well defined, an issue we discussed above when reflecting on the findings of our extensions. The bracketing literature, like Read *et al*. [1], often dichotomizes the continuum of bracketing into either broad or narrow bracketing. However, it is unclear when a certain choice set can be categorized as broad or narrow, or when a change in the choice set or its presentation is considered broader or narrower.

We therefore call for researchers to do further work to reach a cohesive framework that marks a clear, definitive and universally acknowledged definition of choice bracketing, ‘narrow’ and ‘broad’ in decision-making literature. Clarifying the scope and the locus of focus can facilitate greater consistency and clarity in research efforts and enhance efficiency in generating meaningful insights into the decision-making process.

We believe that addressing these conceptual ambiguities can start by conducting systematic reviews and/or meta-analyses of the bracketing and adjacent literatures to map, define, consolidate and identify gaps and discrepancies.

## 15. Limitations

This is an unusual replication, in that we aimed to replicate a set of studies from a review article, rather than conducting a replication of empirical papers. That posed several unique challenges. One of the major challenges was in the very brief descriptions of the studies reviewed, sometimes with no description of the methods and procedures, and without fully reporting its findings, lacking descriptives or statistical information. Some of the studies we replicated from Read and colleagues [1] were only ever described in their review, and so we had no access to needed additional information regarding the studies’ procedures, methods and design to allow for a closer reproduction. We did our best to reconstruct whatever we could from that target article, yet we believe that each of these studies and the ideas it represents is deserving of a full empirical paper that would explore those directions in greater detail and provide more comprehensive information.

Using a questionnaire as a data collection process facilitates conducting surveys with a large sample size. However, decision-making studies aim to study everyday decision-making mechanisms in real-life situations, and surveys can only attempt to synthesize real-life scenarios with word depictions in the question prompts. Hence, the sense of stress, anxiety, pressure or other elements affecting our decision-making behaviour cannot be replicated in this context. This limited immersion restricts the generalizability of the study, and the extent of bracketing reported here may show differences from real-life situations. Hence, given the successful replication of these studies, a natural next step would be for future studies to try and more accurately resemble real-life situations by implementing experimental designs incorporating immersive simulations, decision tasks and/or in the field to examine more realistic everyday-life decision-making.

## 16. Conclusion

We conducted a close replication of seven studies reviewed in Read *et al*. [1] and based on a pre-registered criteria concluded an overall successful replication with support for six out of the seven studies.

## Data Availability

All materials, data and code are publicly available on: [49]. Supplementary material is available online [50].

## Footnotes

^1^
We note that our Stage 1 had a pre-registration inconsistency. In our Stage 1 table 6, we wrote in the table note that participants who chose ‘Reading a driver’s manual in preparation for a driver’s exam’ and/or ‘Planting flower bulbs’ would be excluded from the analyses. Yet in this section (‘assumption checks (extension)’) we wrote that we will conduct these exclusions ‘in case we fail to find support for Study 3’. The differences in the findings comparing with and without exclusions were minor and are provided with our Rmarkdown output (with exclusions: *V* = 0.66; without: *V* = 0.65). In our §12 below, we follow the earliest pre-registered comment in our Stage 1 table 6 and report the results with exclusions.
